# Synaptic Zinc: An Emerging Player in Parkinson’s Disease

**DOI:** 10.3390/ijms22094724

**Published:** 2021-04-29

**Authors:** Joanna Sikora, Abdel-Mouttalib Ouagazzal

**Affiliations:** 1 CNRS, Laboratoire de Neurosciences Cognitives, (UMR 729), Aix Marseille Université, 13331 Marseille, France; joanna.sikora@u-bordeaux.fr; 2 CNRS, Institut des Maladies Neurodégénératives, (UMR 5293), Université de Bordeaux, 33000 Bordeaux, France

**Keywords:** synaptic zinc, NMDA receptors, striatum, motor behavior, mice, Parkinson’s disease

## Abstract

Alterations of zinc homeostasis have long been implicated in Parkinson’s disease (PD). Zinc plays a complex role as both deficiency and excess of intracellular zinc levels have been incriminated in the pathophysiology of the disease. Besides its role in multiple cellular functions, Zn^2+^ also acts as a synaptic transmitter in the brain. In the forebrain, subset of glutamatergic neurons, namely cortical neurons projecting to the striatum, use Zn^2+^ as a messenger alongside glutamate. Overactivation of the cortico-striatal glutamatergic system is a key feature contributing to the development of PD symptoms and dopaminergic neurotoxicity. Here, we will cover recent evidence implicating synaptic Zn^2+^ in the pathophysiology of PD and discuss its potential mechanisms of actions. Emphasis will be placed on the functional interaction between Zn^2+^ and glutamatergic NMDA receptors, the most extensively studied synaptic target of Zn^2+^.

## 1. Introduction

Zinc is the second most prevalent trace element in the human body after iron and is essential for a wide variety of physiological functions. The brain contains a significant amount of zinc, estimated at around 1.5% of the total body content. Cellular zinc level is tightly controlled by the cooperative function of the number of zinc transporters (ZnTs and ZIPs) and metallothioneins that regulate its uptake, efflux and storage in organelles and synaptic vesicles [1]. Intracellular zinc can be divided into three major pools in the brain: (1) a static pool that represents approximately 80% of the total amount of zinc in the brain and consists of structural and catalytic zinc bound to metalloenzymes and metalloproteins; (2) a mobilized pool that comprises labile Zn^2+^ stored in synaptic vesicles and intracellular organelles; and (3) a very small portion of labile Zn^2+^ kept at a very low level in the cytosol [2,3]. Despite its importance in cellular function, excess of labile or ionic zinc (Zn^2+^) is cytotoxic and has been incriminated in a wide variety of neurological disorders from ischemic stroke to neurodegenerative diseases. Alterations of intracellular zinc homeostasis are now recognized as a key factor in the development of PD. Both deficiency and excess of intracellular zinc levels have been implicated in the development of the disease, though overwhelming evidence favor the later mechanism. Besides, it is a key role in a myriad of cellular processes, Zn^2+^ also acts as a synaptic transmitter in the brain and emerging evidence now indicates that alterations of vesicular (or synaptic) Zn^2+^ signaling in basal ganglia may also contribute to PD. In this review, we will provide a brief update on the link between intracellular zinc dyshomeostatsis and PD and cover recent evidence that substantiate the role of synaptic zinc in the pathophysiology of PD. The functional relationship between synaptic zinc and the glutamatergic system will also be addressed with an emphasis on NMDA receptors (NMDAR), the best characterized synaptic target of Zn^2+^.

## 2. Zinc Dyshomeostasis and Parkinson’s Disease

Zinc and other heavy metals have received considerable attention in neurodegenerative diseases because of their cytotoxicity. The role of zinc in the pathogenesis of PD is not straightforward because of its numerous and complex function. Both deficiency and excess of zinc have been incriminated in the development of the disease, though overwhelming evidence favor the later mechanism. 

### 2.1. Zinc Deficiency and Parkinson’s Disease

Several studies have examined whether plasma and CSF zinc levels are altered in PD patients. The reported data are, however, disparate. In some studies, circulating zinc levels were lower in PD patients [4,5,6,7,8,9,10,11], while in others they were normal or even increased [12,13,14,15,16,17,18]. Recent meta-analysis studies, though, point to lower zinc levels in serum and plasma and CSF of PD patients compared to healthy controls [19,20]. Findings from epidemiological studies examining the association of dietary intake of zinc and PD are also contradictory. Higher intake of zinc was associated with reduced risk of PD in some studies [21], but negative findings were reported by others [22,23]. The association between lower levels of circulating zinc and PD has been explained by its antioxidant role since this trace element is essential for a variety of enzymes and proteins (superoxide dismutase oxidative, metallothioneins, and interleukins) involved in oxidative stress and inflammation [24]. In support of this view, animal studies showed that exogenous zinc can produce its beneficial effects by multiple mechanisms. In vitro, zinc inhibits 6-OHDA-induced oxidative stress [25] and reduces methamphetamine-induced dopaminergic neurotoxicity by the increasing expression of metallothioneins, which in turn prevent the generation of reactive oxygen species [26,27]. Zinc treatment also reduces *α*-synuclein (α-syn), the predominant component of Lewy bodies, induced by methamphetamine in cell culture [28]. Finally, zinc deficiency has been suggested to lead to dysfunction of PARK2 (E3 ligase) that possesses zinc-binding domains. PARK2 binds eight zinc ions and the removal of zinc causes a near-complete unfolding of the protein and, thereby, loss of its function [29]. Accordingly, supplementation with zinc has been shown to increase lifespan, as well as motor function in the parkin KO Drosophila model of PD [30].

### 2.2. Zinc Excess and Parkinson’s Disease

There is an overwhelming body of evidence implicating an excess of ionic Zn^2+^ in dopaminergic neurodegeneration associated with PD. Zinc exposure has been identified as an environmental risk factor for PD [31] and post-mortem studies revealed excessive zinc depositions in the substantia nigra (SN) and the striatum of patients with idiopathic PD [13,32,33,34,35]. In line with these observations, in vitro and in vivo experiments with animal models of PD showed that cytosolic accumulation labile zinc is a hallmark of degenerating dopaminergic neurons [36,37,38,39,40,41,42]. Importantly, treatments with intracellular zinc chelators prevent neurodegeneration caused by many neurotoxins (6-OHDA, MPTP, and paraquat) confirming that cytosolic Zn^2+^ accumulation contributes to dopaminergic neuronal loss [43,44,45]. The mechanisms responsible for Zn^2+^ accumulation in the SN of PD patients are poorly understood. One possible cause could be a failure of the intracellular mechanisms that maintain Zn^2+^ homeostasis. In this respect, the human PARK9 (ATP13A2), a lysosomal type 5 P-type ATPase associated with autosomal recessive early-onset PD, has been shown to act as a transporter for lysosomal sequestration of cytoplasmic zinc [46,47,48]. In vitro, loss of PARK9 function causes an imbalance of zinc intracellular homeostasis that in turn leads to lysosomal impairment, accumulation of α-syn, and mitochondrial dysfunction [46,47,48].

More recently, Tamano and colleagues showed that increased cytosolic levels of toxic Zn^2+^ can also be caused by the influx of extracellular zinc into dopaminergic neurons [39,40,41]. In brain slices, both 6-OHDA and paraquat have been found to rapidly increase intracellular Zn^2+^ levels only in the SNc. The increase in intracellular Zn^2+^ concentrations was linked to the entry of extracellular Zn^2+^ through AMPA receptors because it could be blocked by CaEDTA and by an AMPA receptor antagonist (CNQX). Furthermore, combined infusions of intracellular Zn^2+^ chelators (ZnAF-2DA, TPEN) and 6-OHDA or paraquat into the SNc reduce the loss of nigrostriatal dopaminergic neurons and the associated motor deficits in rats. 

While the above studies implicate dyshomeostasis of intracellular Zn^2+^ pool in dopaminergic cell loss, several studies showed that systemic or intra-nigral injections of exogenous zinc can, on its own, produce dopaminergic neurotoxicity [36,37,49,50,51]. For instance, chronic injections of zinc systemically cause degeneration of the nigrostriatal dopaminergic pathway and locomotor deficits in rats like the pesticide, paraquat [52,53,54]. Zinc treatment causes cellular dysfunction by increasing oxidative stress through activation of nicotinamide adenine dinucleotide phosphate (NADPH) oxidase and depletion of glutathione (GSH), which in turn trigger the apoptotic machinery leading to neuronal loss, as seen following paraquat treatment [36]. The motor deficits and dopaminergic cell neurodegeneration induced by chronic zinc treatments were also shown to involve activation of microglial cells and expression of inflammatory mediators (e.g., TNF-α, IL-1β) [55,56]. 

Viewed together, the evidence suggests that endogenous Zn^2+^ is a key actor in the pathophysiology of PD. However, the role of this cation seems highly complex as both beneficial and deleterious actions of intracellular Zn^2+^ have been implicated in PD. Beneficial actions have been linked to Zn^2+^ role in protection against oxidative stress by influencing the activity of antioxidant enzymes and signaling pathways, while deleterious actions have been attributed to generation of oxidative stress caused by intracellular Zn^2+^ overload. Beside alterations of intracellular zinc pools, emerging evidence now suggests that dysfunction of synaptic Zn^2+^ signaling may also contribute to PD. 

## 3. Synaptic Zinc in the CNS, Zinc Staining Methods and Gluzincergic Neurons

The first evidence for the presence of labile Zn^2+^ or chelatable Zn^2+^ in the brain was provided more than 6 decades ago by Maske and co-workers (1955) who revealed focal deposits of Zn^2+^ in the hippocampus using an auto-metallographic technique (dithizone staining procedure) [57]. Subsequent studies combining Timm’s sulfide silver staining method with electron microscopy demonstrated that chelatable Zn^2+^ deposits were mainly localized within synaptic vesicles of hippocampal mossy fibers [58,59]. The notion of vesicular or synaptic zinc was then introduced by Turner McLardy in 1970 [60] and zinc-enriched neurons have been proposed to be called zincergic neurons. Most of the above studies were, however, performed by the original staining method of Timm [61] that was not specific for ionic zinc. In the early eighties, zinc-specific staining methods were developed by Danscher (Timm and Danscher or NeoTimm method) for visualizing zinc-positive neuronal terminals [62], as well as zinc-positive neurons using retrograde axon tracing [63]. Recently, numerous membrane-permeable fluorophores (e.g., Zinpyr, TSQ, Zinquin) with high selectivity for ionic zinc were also developed for mapping zincergic pathways in the brain and studying zinc dynamics at the synaptic level [64,65].

**Figure 1:**
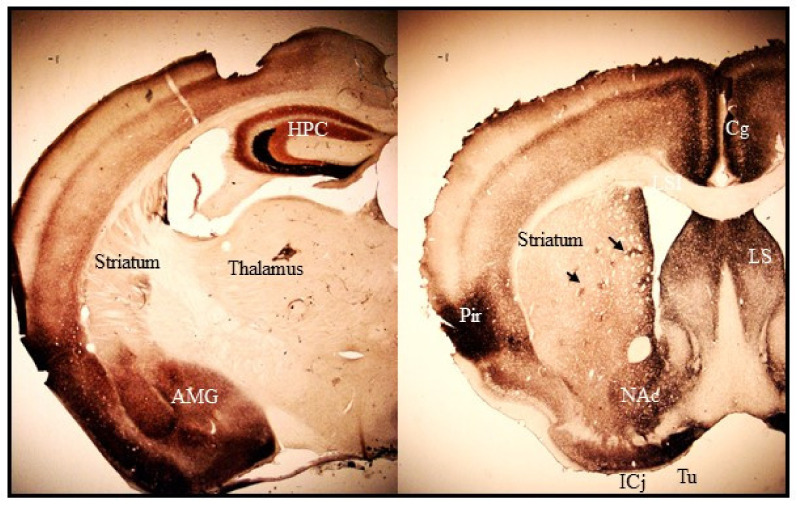
Photomicrographs of coronal sections of mouse brain representing synaptic Zn^2+^ staining in cortico-limbic and striatal regions. The tan-brown-black staining corresponds to histochemically reactive Zn^2+^ revealed with the NeoTimm staining method. Abbreviations: HPC, hippocampus; AMG, amygdala complex; Cg, cingulate cortex; Pir, piriform cortex; LS, lateral septal nucleus; NAc, nucleus accumbens; ICj, island of Calleja; Tu, olfactory tubercle. Black arrows indicate striatal zinc-positive patches.

Histochemically reactive zinc is found mostly in the forebrain regions. The heaviest staining is present in the hippocampus, amygdala, cerebral cortex, and olfactory bulb where zincergic neurons are located (Figure 1). The striatum, septum and bed nucleus of the stria terminalis, which only receive afferent zincergic connections, show moderate staining (Figure 1, [66]). Outside the forebrain, faint staining is present in the cerebellum and spinal cord [66,67]. The pattern of zinc staining overlaps closely with the expression profile of ZnT3 protein, which is up to date the sole known zinc transporter protein responsible for loading Zn^2+^ into synaptic vesicles. Knockout (KO) mice lacking the ZnT3 gene have no histochemically reactive zinc in the brain revealed by NeoTimm staining [68] or zinc-binding fluorescent dyes [69].

Most synaptic zinc in the CNS is found in neurons that use glutamate as the main neurotransmitter. Studies combining NeoTimm staining with electron microscopy showed that zinc-positive boutons in the forebrain make asymmetric synaptic contacts on dendritic spines and are opposed to post-synaptic membranes enriched in glutamatergic receptors [70]. However, not all glutamatergic neurons contain zinc in their synaptic vesicles [23,26,27]. Gluzinergic neurons represent only a subset of the broader set of all glutamatergic neurons. Synaptic zinc is also found in GABAergic neurons within the cerebellum and in GABAergic and glycinergic neurons in the spinal cord [71].

### Synaptic Zinc and Glutamatergic System

Early evidence supporting vesicular zinc release comes from studies reporting the disappearance of Timm’s staining in mossy fibers upon electrical stimulation or ischemic insult [72,73,74,75]. Subsequent micro-dialysis studies in rats and rabbits showed a transient increase in extracellular zinc level following chemical and electrical stimulation of mossy fiber axons [76,77]. More recent studies combining zinc imaging with fluorescent extracellular zinc indicators (FluoZin-3) and electrophysiological recordings in the hippocampus provided direct evidence of zinc release from synaptic terminals into synaptic space upon depolarization [78,79]. Comparable experiments conducted on ZnT3 knockout confirmed the vesicular origin of released zinc [80,81]. 

Upon its release, synaptic Zn^2+^ can act on a myriad of targets as numerous synaptic receptors and voltage-gated calcium channels possess binding sites with a nanomolar affinity for extracellular zinc. In vitro, Zn^2+^ inhibits the activity of glutamate, GABA_A_, glycine, and cholinergic receptors. It also blocks the activity of voltage-gated calcium channels, as well as sodium, potassium, and chloride channels [82]. Zn^2+^ can also translocate into postsynaptic neurons and act as a transcellular messenger, adding other layers of complexity. Upon its release, Zn^2+^ can enter postsynaptic neurons through several ion channels and modulate the activity of a variety of mitogen-activated protein kinase (MAP Kinase) pathways in response to extracellular stimuli [83,84].

Among synaptic targets of Zn^2+^ currently known, NMDA receptors (NMDAR) have been the most extensively studied because of their exquisite sensitivity to this cation. NMDARs exist as multiple subtypes that vary in their biophysical, pharmacological, and signaling properties. NMDARs are heteromeric complexes composed of 4 subunits derived from 3 families: GluN1, GluN2 (N2A-D), and GluN3 (N3A-B). The GluNR1 combines with GluN2 or GluN3 subunits to form a functional receptor [85,86]. NMDARs are calcium-permeable channels that require binding of both glutamate and glycine (or D-serine) for full activation. At resting state, the channel is gated by Mg^2+^ that is extruded during depolarisation allowing cations to enter. NMDARs also possess several modulatory sites that affect receptor function. These include the polyamine site, Zn^2+^ site, proton-sensitive site, and a redox modulatory site [85,86]. 

Zinc inhibits NMDA receptors in voltage-independent and voltage-dependent manners. At high concentrations, zinc binds to the magnesium site inside the channel pore and causes voltage-dependent inhibition of NMDAR current [87]. At low concentrations, zinc causes voltage-independent non-competitive inhibition of NMDARs by acting at the high-affinity binding site located at the N-terminal domain of GluN2 subunits. The inhibition mediated by GluN2 subunits is a purely allosteric mechanism: zinc-binding reduces channel opening probability (gating kinetics) but not its conductance. NMDAR sensitivity to the allosteric inhibition by zinc strongly depends on the GluN2 subunit composition. Receptors containing the GluN2A subunit are exquisitely sensitive to extracellular zinc. Zn^2+^ IC50 concentrations are of nanomolar range (a high-affinity allosteric inhibition) for NR1/GluN2A receptors, sub-micromolar range (~2 μM, a low-affinity allosteric inhibition) for NR1/GluN2B receptors and high micromolar range (≥10 μM) for NR1/GluN2C and NR1/GluN2D receptors [87,88,89,90]. Such difference in zinc affinities implies that GluN2A-NMDARs are the major targets of synaptically released zinc. Accordingly, Vergnano et al. (2014) showed that in the hippocampus the high-affinity GluN2A zinc-binding site is the primary target of vesicular zinc and that zinc concentrations in the synaptic cleft are unlikely to impact the micromolar binding sites of NMDARs [91]. They found that under basal conditions, ambient extracellular levels of zinc are not sufficiently high to occupy the high-affinity (nM) zinc-binding sites and cause tonic inhibition of synaptic NMDARs in hippocampal slices (see also [92,93]). However, upon application of brief repetitive synaptic stimulations, zinc transiently rises in the synaptic cleft and inhibits postsynaptic GluN2A-NMDARs but not GluN2B-NMDARs. A key feature of GluN2A-high-affinity zinc inhibition is that it is not total, as 20–40% of the maximal NR1/NR2A currents remains at saturating zinc concentrations [88,94]. More recently, the physiological relevance of Zn^2+^ action on GluN2A-NMDARs has been demonstrated in vivo using knock-in (KI) mice carrying a point mutation (GluN2A-H128S) in the zinc-binding site of the GluN2A subunit [95]. In vitro, GluN2A-H128S mutation selectively eliminates high-affinity (nanomolar) zinc inhibition of NMDARs [95] and enhances NMDAR-dependent long-term potentiation (LTP) in hippocampal slices [91]. In vivo, the mutation enhances basal pain responses to radian heat and capsaicin but not to mechanical or thermal noxious stimuli suggesting that synaptically released zinc acting in the GluNR2A subunit modulates specific aspects of pain processing [95]. It also improves contextual fear learning [96] and acquisition of new motor skills (See Section 5.3 below).

## 4. Synaptic Zinc in the Basal Ganglia

Few studies have closely examined synaptic zinc distribution within the basal ganglia circuits. The striatum shows a moderately high level of histochemically reactive Zn^2+^ as revealed by NeoTimm staining methods (Figure 1). The subthalamic nucleus that contains excitatory glutamatergic neurons is largely devoid of histochemically reactive zinc. NeoTimm staining is also absent in the globus pallidus and substantia nigra, which receive excitatory glutamatergic projections from the cortex and subthalamic nucleus [97,98,99,100]. The striatum is, therefore, the sole region within the basal ganglia where the presence of histochemically reactive zinc was consistently revealed in numerous vertebrate species [99,101,102,103].

### Striatal Synaptic Zinc

Synaptic zinc staining in the dorsal striatum (caudate-putamen) shows a heterogeneous distribution. Heavy staining is present at the peripheral rim of the medial, dorsal, and lateral regions along the rostro-caudal axis [99,102]. In the inner region, intensely stained patches are frequently visible (Figure 1). The distribution of the zinc-rich patches has been shown to somewhat follow the established striosome-matrix compartmentalization [99,102]. However, the shape of zinc-rich patches does not coincide with the delineation of striosomal compartment obtained with immunohistochemical staining for calbindin-D28K or acetylcholinesterase. They are smaller and more irregular than striosomes [99]. No zinc staining is found in myelinated fiber bundles within the striatum. Heterogeneous staining for reactive zinc is also observed in the ventral part of the striatum and the nucleus accumbens, though the intensity of the staining is denser than in the dorsal striatum (Figure 1, [99]). 

While a high density of synaptic zinc is observed in the striatum, no zincergic neurons are found in this brain region indicating that zinc-positive terminals derive from extra-striatal sources [102,104]. Few studies have investigated in detail the origins of zinc-positive boutons in the striatum in rats using lesion techniques (transection of the corpus callosum, intra-cortical infusion of colchicine, and electrolytic lesion of the amygdala) and Fluoro-Gold tracing coupled with histochemical staining of zincergic neurons and terminals. Cortical gluzinergic neurons projecting to the striatum are located throughout the neocortex, but the major proportion is found in the frontal motor cortex areas [102]. They are also found in the amygdala, a limbic structure that heavily projects to the ventral part of the striatum and the nucleus accumbens [23,65,68]. On the other hand, glutamatergic projections from the thalamus and ventral hippocampus into the striatum are devoid of synaptic Zn^2+^ [66,102].

Little is known about synaptic Zn^2+^ contribution to striatal synaptic transmission. Experiments performed with exogenous zinc show that major striatal neurons are sensitive to the action of this cation [52,53,105,106]. In striatal slices, Zn^2+^ reduces the excitatory responses of striatal neurons evoked by stimulations of the sensorimotor cortex [43]. Zn^2+^ was also shown to directly inhibit basal activity of GABAergic medium spiny neurons (MSN), the principal population of projection neurons of the striatum, and blocks depolarization of MSNs induced by substance P and a neurokinin 1 (NK1) receptor agonist [52]. On the other hand, it enhances the excitability of cholinergic interneurons (CIN) by blocking leak K^+^ channels (TASK-3, [105]). Zinc also disinhibits CINs by reducing postsynaptic GABAA currents [106].

## 5. Synaptic Zinc and Parkinson’s Disease

The degeneration of nigrostriatal dopaminergic neurons results in overactivation of glutamatergic projections to the striatum that is considered a key mechanism contributing to the development of the clinical symptoms and neurodegeneration in PD [44,107]. The abundance of vesicular Zn^2+^ in cortico-striatal pathways suggests that synaptically released Zn^2+^ alongside glutamate may also play a role in PD. In the following section, we will introduce our recent work addressing the involvement of synaptic Zn^2+^ in dopaminergic neurotoxicity and behavioral deficits of PD and discuss its potential mechanisms of actions.

### 5.1. Effects of Synaptic Zinc Elimination on 6-OHDA Neurotoxicity

**Figure 2:**
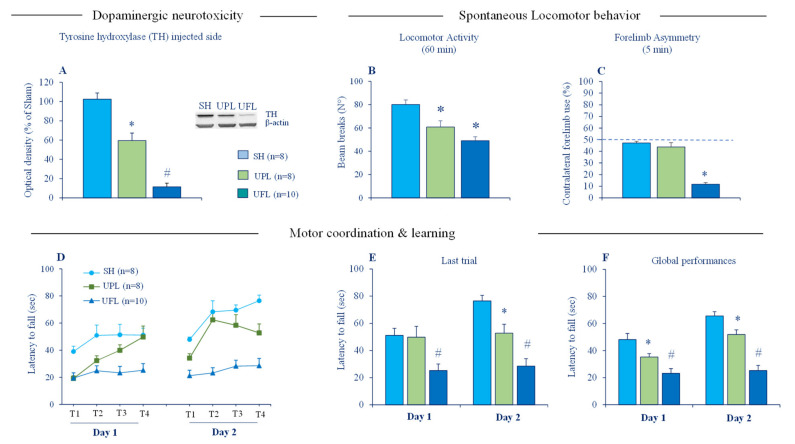
Effects of partial and full unilateral intrastriatal 6-OHDA lesions on striatal tyrosine hydroxylase (TH) protein expression and motor function in C57/BL6 J mice. (**A**) The left panel depicts the densitometric analysis of TH expression in the striatum of WT, unilaterally, partially lesioned (UPL) and unilaterally, fully lesioned (UFL) mice (*n* = 8–10 per group); the right panel depicts a representative blot of TH protein in lesioned striatal sides with β-actin as the reference, western blotting analysis were performed in duplicates of the sample. (**B**) Locomotor activity of mice tested in Actimetry cages for 60 min. (**C**) Contralateral forelimb uses of mice tested in cylinder task over 5 min. The dotted line indicates symmetric use of forepaws (**D**) Mean latencies to fall from the rod during training sessions (S1, S2, S3, and S4) in day 1 and 2 in the accelerated rotarod task. (**E**) Mean latencies to fall from the rod in the 4th training session (last session) in day 1 and 2. (**F**) Mean latencies to fall from rotating rod averaged across all sessions in day 1 and 2. SH: sham (non-lesioned) mice, PUL: mice with partial unilateral intrastriatal 6-OHDA lesion and FUL: mice with a full unilateral intrastriatal 6-OHDA lesion. Data expressed as mean ± SEM. * *p* < 0.05 vs. Sham group, # *p* < 0.05 vs. all groups, Fisher’s PLSD post-hoc test following a significant one-way ANOVA.

Cytosolic accumulation of toxic Zn^2+^ is a cardinal feature of degenerating dopaminergic neurons in PD and animal models of PD [45,108,109,110,111] and is thought to be a key pathogenic mechanism underlying dopaminergic cell death. Excessive accumulation of toxic Zn^2+^ has been linked to release from intracellular stores (lysosomes, mitochondria and metallobinding proteins), as well as an influx from the extracellular milieu [39]. In normal conditions, no detectable histochemically reactive Zn^2+^ staining is seen in the SN [66] but labeled ^65^Zn infused into the striatum has been demonstrated to undergo anterograde transport to the SN [112]. Intrastriatal infusions of Zn^2+^ either alone or in combination with DA was also reported to induce degeneration of the nigrostriatal dopaminergic pathway in rats [109]. Considering the hyperactivity of cortico-striatal pathways in PD, it is therefore, possible that Zn^2+^ released in excess alongside glutamate may enter injured dopaminergic terminals and exacerbates ongoing neurotoxicity. We directly tested this idea by assessing the susceptibility of ZnT3 KO mice to neurotoxic effects of 6-hydroxydopamine (6-OHDA). However, no hint of neuroprotection was detected at a cellular level. ZnT3 KO and WT mice subjected either to partial unilateral or bilateral intrastriatal 6-OHDA lesion displayed a comparable loss of striatal tyrosine hydroxylase (TH), the specific marker of dopaminergic fibers [113]. The partial intrastriatal 6-OHDA lesion model we used mimics the early stages of PD and has many advantages. It produces a moderate striatal DA depletion and mild motor impairments in mice (Figure 2), making it possible to detect changes in both directions, potentially beneficial and deleterious effects of synaptic zinc elimination. It is also suitable for revealing a potential neurotoxic role of synaptic Zn^2+^ because 6-OHDA and Zn^2+^ induce their cytotoxicity through comparable mechanisms involving the generation of reactive oxygen species (ROS), as well as mitochondrial dysfunction and energy failure [54,114]. The absence of neuroprotective phenotype in ZnT3 KO mice clearly shows that vesicular Zn^2+^ is not necessary for 6-OHDA neurotoxicity. It should be stressed that neither partial nor full 6-OHDA lesion, which mimics advanced PD, changed the expression of ZnT3 protein in the striatum [113]. By contrast, a clear-cut upregulation of striatal VGluT1 protein, the presynaptic marker of cortico-striatal glutamate terminals, was detected after the full 6-OHDA lesion. Such dichotomy in the effect of DA depletion on the expression of striatal VGlut1 and ZnT3 implies that regulation of vesicular storage and release of glutamate and Zn^2+^ may be uncoupled in the context of PD.

### 5.2. Effects of Synaptic Zinc Elimination and Extracellular Zinc Chelation on Motor Deficits of Intrastriatal 6-OHDA Lesions

**Figure 3:**
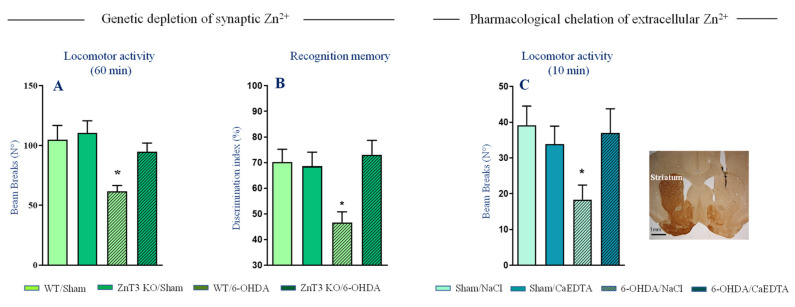
Genetic elimination of vesicular Zn^2+^ and intrastriatal chelation of extracellular Zn^2+^ improve behavioral deficits of 6-OHDA lesions. (**A**) Locomotor activity of ZnT3 KO and WT mice subjected to partial unilateral intrastriatal 6-OHDA lesion (*n* = 7–8 per genotype). (**B**) Recognition memory of ZnT3 KO and WT mice subjected to partial bilateral intrastriatal 6-OHDA lesion (*n* = 7–10 per genotype). Corresponding control groups, Sham/ZnT3 KO and Sham/WT mice received intrastriatal injection of vehicle (*n* = 7–12 per genotype). (**C**) Locomotor activity of BL6J mice subjected to full unilateral intrastriatal 6-OHDA lesion. Mice received CaEDTA (Sham/CaEDTA and 6-OHDA/CAEDTA, *n* = 7 per treatment) or vehicle (Sham/NaCl and 6-OHDA/NaCl, *n* = 6–7 per treatment) injection into dorsal striatum. Representative coronal section showing a complete loss of TH-positive fibers and injector needle placement in the dorsal striatum. Data expressed as mean ± SEM, * *p* < 0.05 vs. Sham/WT or Sham/NaCl group, Dunnett’s post-hoc test following a significant two-way ANOVA. Modified with permission from Sikora et al., (2020) [113].

Another way vesicular Zn^2+^ may play a role in PD is by altering synaptic transmission and thus promoting the expression of motor deficits. Studies evaluating behavioral effects of intrastriatal zinc infusions showed that enhanced extracellular Zn^2+^ levels in the striatum impairs locomotor activity in rats [115,116,117]. Whether such deleterious effects are due to alterations of synaptic transmission and neurotoxicity caused by repeated Zn^2+^ injections are unclear. In control conditions, synaptic Zn^2+^ does not seem however to suppress spontaneous locomotor behavior. Numerous studies [118,119] including ours (Figure 3A, [113]) reported a normal baseline locomotor activity and exploratory behavior in ZnT3 KO mice (but see also [120]). On the other hand, we found that ZnT3 KO mice are remarkably resistant to locomotor deficits and memory impairment induced by partial 6-OHDA lesions (Figure 3A,B), indicating that synaptically released Zn^2+^ facilitates expression of behavioral deficits. Accordingly, acute chelation of extracellular Zn^2+^ directly in the striatum restored locomotor activity impairment induced by full 6-OHDA lesion (Figure 3C). 

Taken together, these findings provide strong evidence for a role of synaptic Zn^2+^ in the pathophysiology of PD and suggest that synaptically released Zn^2+^ in the striatum may promote behavioral deficits by altering synaptic transmission. How synaptic Zn^2+^ is recruited upon DA depletion and mediates its deleterious actions is unclear. The lack of effect of 6-OHDA lesion on striatal ZnT3 level and the absence of neuroprotective phenotype in lesioned ZnT3 KO mice argues again an increase in vesicular Zn^2+^ storage and release. In striatal slices, exogenous Zn^2+^ alters the activity of a variety of receptors (e.g., NMDA, GABAA, NK1, nicotinic receptors) and ion channels (acid-sensing ion channels (ASIC), ClC-2 type chloride channel, leak K+ channels) [69,70,72]. Given the profound structural changes that take place within striatal circuitry upon DA depletion, it is thus possible that many of these targets may become readily accessible to modulation by vesicular Zn^2+^. In such situation, synaptically released Zn^2+^ may exert both beneficial and deleterious modulatory actions depending on the synaptic targets and their localizations, but the net result being detrimental at the behavioral level. 

### 5.3. Effects of Abrogation of Zinc Action on GluN2A-NMDARs on Motor Deficits of Intrastriatal 6-OHDA Lesion

NMDARs stand out for their high sensitivity to Zn^2+^ and they play a prominent role in PD. NMDARs overactivation is a key feature of PD and they are a target for the development of antiparkinsonian and neuroprotective agents (e.g., amantadine and memantine) [121]. In PD, the subunit composition of the NMDARs does not remain static but changes depending on the degree of DA depletion. For instance, a full striatal DA lesion reduces GluN2B subunit but not GluN2A expression. On the other hand, partial striatal DA lesion increases GluN2A level and reduce NMDAR-dependent cortico-striatal synaptic plasticity. Pretreatment with TAT2A peptide, which downregulates the expression of synaptic GluN2A-NMDA receptors, normalizes LTP and improves motor deficits of partial DA lesion [107,122]. These observations highlight the dynamic regulation of NMDAR subunits and the profound consequences it has on the functioning of synapses and networks in the context of striatal DA depletion. They also raise important questions about whether and how Zn^2+^ modulation of GluN2A-NMDA receptors impacts motor function in normal and PD conditions. 

**Figure 4:**
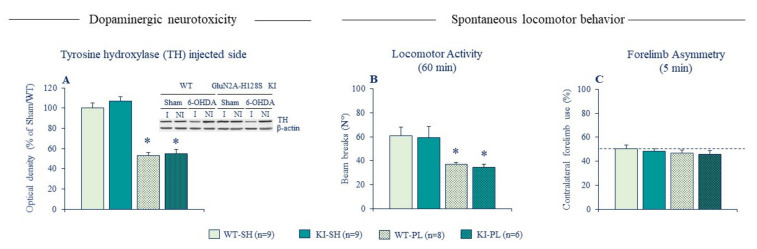
Ablation of zinc-GluN2A binding site has no impact on neurotoxicity and locomotor deficits of partial unilateral intrastriatal 6-OHDA lesion. (**A**) The left panel depicts the densitometric analysis of TH protein expression in the striatum of sham and lesioned male GluN2A-H128S KI mice and WT littermates (*n* = 6–9 per genotype); western blotting analysis were performed in duplicates of the sample. The upper right panel depicts a representative blot of TH protein of injected (I) and non-injected (NI) striatal sides with β-actin as the reference. (**B**) Locomotor activity of sham and lesioned KI mice and WT counterparts tested in Actimetry cages. (**C**) Contralateral forelimb use of sham and lesioned male GluN2A-H128S KI mice and their WT counterparts in cylinder test. The dotted line indicates symmetric use of forepaws. Data expressed as mean ± SEM. * *p* < 0.05 vs. Sham/WT group, Fisher’s PLSD post-hoc test following a significant two-way ANOVA.

To address these issues, we used GluN2A-H128S KI mice that lack the high-affinity zinc inhibition of GluN2A-NMDARs. Behavioral phenotyping of these mice revealed no alterations in baseline locomotor exploration (Figure 4, [95]), again confirming the absence of intrinsic synaptic Zn^2+^ tone modulating spontaneous locomotor behavior in control conditions. KI mice displayed also a normal susceptibility to neurotoxicity and locomotor effects of partial 6-OHDA lesion, unlike ZnT3 KO mice that were resistant to behavioral deficits of the lesion. These findings were initially reported in female mice [113] and replicated in a recent follow-up study in male mice (Figure 4). The absence of phenotype in lesioned KI mice implies that synaptic Zn^2+^ promotes locomotor deficits of DA lesion through NMDAR-independent mechanisms. Synaptically released Zn^2+^ may act both extracellularly and intracellularly to mediate its deleterious actions. One plausible mechanism could be by exacerbating the overactivation of striatal cholinergic transmission. Aberrant plasticity in CINs and increased cholinergic signaling in the striatum is considered a key mechanism underlying the expression of PD motor deficits [123,124,125,126]. As noted earlier, Zn^2+^ increases CIN activity by directly blocking leak K^+^ channels (TASK-3, [105]) and GABA_A_ receptors [106]. By acting on the former targets, synaptically released Zn^2+^ may contribute to the abnormal increase in striatal cholinergic signaling, which in turn exacerbates locomotor deficits of DA depletion.

**Figure 5:**
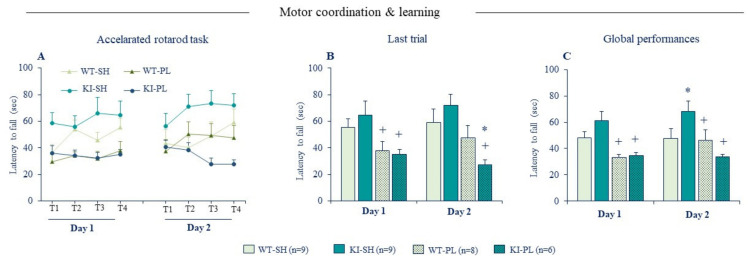
Ablation of zinc-GluN2A binding has opposite effects on motor learning in sham and 6-OHDA lesioned mice. (**A**) Mean latencies to fall from the rotating rod during training sessions (S1, S2, S3, and S4) in day 1 and 2 in the accelerated rotarod task. (**B**) Mean latencies to fall from rotating rod in the 4th training session (last session) in day 1 and 2. (**C**) Mean latencies to fall from rotating rod averaged across all sessions in day 1 and 2. SH: sham (non-lesioned) mice and PL partial unilateral intrastriatal 6-OHDA lesion (*n* = 6–9 per genotype). Data expressed as mean ± SEM. * *p* < 0.05 vs. Sham/WT group, + *p* < 0.05 vs. Sham/KI group, Fisher’s PLSD post-hoc test following a significant two-way ANOVA.

Considering the prominent role of GluN2A-NMDARs in PD and its high sensitivity to Zn^2+^, we used the accelerated rotarod task to characterize further the effects of partial 6-OHDA lesion on motor learning. Acquisition of new motor skills recruits NMDAR-dependent synaptic plasticity in the dorsal striatum [127] and genetic deletion or pharmacological blockade of striatal NMDAR function impairs learning performances in a range of motor tasks [127,128,129]. Interestingly, ablation of Zn^2+^-GluN2A biding site has an opposite effect on motor learning in normal and lesioned mice (Figure 5). Non-lesioned KI mice displayed a good learning performance compared to WT littermates indicating that disinhibition of GluN2A-NMDARs enhances acquisition of new motor skills. Lesioned WT mice exhibited a clear-cut motor coordination/learning deficit in the first training day, but their learning performances improved the second day with continued training and reached those of sham WT mice. Lesioned KI mice were comparable to lesioned WT in the first training day, however they showed no learning improvement on the second day (Figure 5), as seen with the full 6-OHDA lesion (Figure 2D–F). This indicates that loss of Zn^2+^ inhibition of GluN2A-NMDARs exacerbates motor learning deficit caused by the partial DA lesion. The later findings extend those reported by Fantin et al., (2008) [130] in rats showing that pharmacological blockade of GluN2A-NMDARs directly in the dorsal striatum restores motor learning deficits of 6-OHDA lesion in the rotarod task. 

Collectively, the behavioral phenotypes of KI mice reveal a new molecular mechanism through which synaptic Zn^2+^ exerts a contrasting modulatory action on motor learning in normal and PD conditions by dampening GluN2A-NMDAR activity. While Zn^2+^ inhibition of GluN2A-NMDARs has a deleterious consequence on motor learning in a control condition, in the context of striatal DA depletion it has on the contrary a beneficial effect because the aberrant increase in NMDAR signaling underlies motor learning deficits. The fact that GluN2A-H128S mutation did not impact spontaneous locomotor and exploratory behavior implies that Zn^2+^ action on GluN2A-NMDARs may contribute to the modulation of specific aspects of motor function. Such functional role may be explained by the fact that vesicular Zn^2+^ is only present in a subset of cortico-striatal glutamatergic pathways that may particularly be recruited under challenging motor tasks, such as those involving motor skill learning. 

## 6. Conclusions

**Figure 6:**
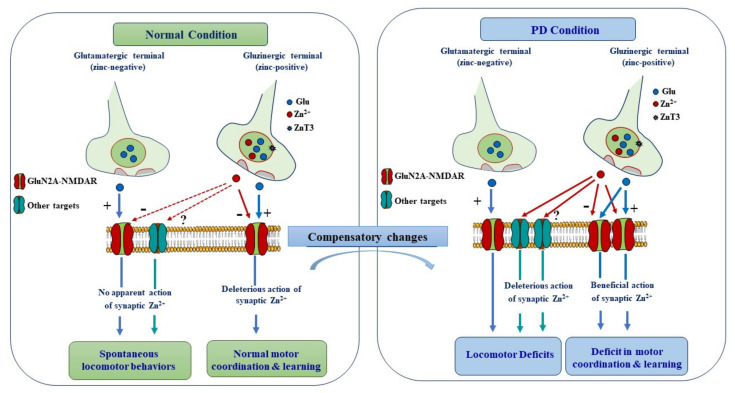
Schematic diagram summering the proposed mechanisms underlying synaptic modulation of motor function in normal and PD conditions. Vesicular Zn^2+^ is present in a subset of cortico-striatal pathways and thus contributes to the modulation of a specific aspect of motor behavior. In normal condition, synaptically released Zn^2+^ from cortico-striatal gluzinergic terminals does not seem to modulate spontaneous locomotor behavior. By contrast, it negatively modulates learning of motor skills by dampening the activity of GluN2A-NMDARs. In PD condition, synaptically released Zn^2+^ is recruited and plays a deleterious role alongside glutamate by promoting the expression of locomotor deficits. However, the detrimental modulatory action of Zn^2+^ is mediated by other targets than NMDARs, which become accessible to Zn^2+^ owing to the profound morphological changes that take place upon DA depletion. In the context of DA depletion, Zn^2+^ action on GluN2A-NMDARs is rather beneficial because the aberrant increase in NMDAR signaling impairs motor learning.

The studies summarized here reveal many facets of synaptic zinc functions and its involvement in PD (Figure 6). They suggest that synaptically released Zn^2+^ from cortico-striatal terminals may predominantly play a deleterious role alongside glutamate by promoting the expression of motor and cognitive deficits associated with PD. Synaptic Zn^2+^ appears to mediate its action mainly by altering striatal synaptic transmission rather than by exacerbating dopaminergic neurotoxicity. The precise mechanisms underlying such deleterious actions remains to be identified. The findings from KI mice clearly indicate that detrimental actions of Zn^2+^ are mediated through NMDAR-independent mechanisms. The inhibitory action of synaptic Zn^2+^ on GluN2A-NMDARs, the most zinc-sensitive glutamatergic receptor, is rather beneficial in the context of DA depletion because NMDAR dysfunction contributes to the development of motor deficits. Excess zinc has been incriminated in dopaminergic neurotoxicity and chelation therapy was proposed as a potential neuroprotective approach in PD [131]. Understanding the mechanisms underlying the detrimental role of the synaptic Zn^2+^ pool may further help to identify novel targets for the development of efficient symptomatic therapies for PD and related disorders.

## Data Availability

Not applicable.
